# 1508. The effects of the COVID-19 pandemic on depression severity and viral load non-suppression among people with HIV accessing care in an urban infectious disease clinic in Baltimore, MD

**DOI:** 10.1093/ofid/ofad500.1343

**Published:** 2023-11-27

**Authors:** Tarfa Verinumbe, Catherine Lesko, Richard Moore, Anthony Fojo, Jeanne Keruly, LaQuita N Snow, Jarratt Pytell, Oluwaseun Falade-Nwulia

**Affiliations:** Johns Hopkins University, Baltimore, Maryland; Johns Hopkins Bloomberg School of Public Health, Baltimore, Maryland; Johns Hopkins University, Baltimore, Maryland; Johns Hopkins University School of Medicine, Baltimore, Maryland; The Johns Hopkins University School of Medicine, Baltimore, MD; Johns Hopkins University, Baltimore, Maryland; University of Colorado, Denver, Colorado; Johns Hopkins University, Baltimore, Maryland

## Abstract

**Background:**

The COVID-19 pandemic heightened concerns about people experiencing more depressive symptoms. Among people with HIV (PWH), who have higher rates of depression, these symptoms may lead to adverse HIV-related outcomes. This study sought to characterize the effects of the COVID-19 pandemic on depression severity and to investigate the association between depression trajectories and viral load (VL) non-suppression among PWH enrolled in HIV care.

**Methods:**

The study sample was PWH in the Johns Hopkins HIV Clinical Cohort who reported depression symptoms on the Patient Health Questionnaire 8 (PHQ-8) via a self-administered survey pre-pandemic (Mar. 1, 2018 - Feb. 28, 2020) and during the COVID-era (Sept. 1, 2020 - Feb. 28, 2022). Depression severity was categorized using standard PHQ-8 cutoffs ranging from normal (0-4) to severe (20-24). Depression severity categories pre-pandemic (last survey) and COVID-era (earliest survey) were compared, and trajectories were classified as: 1) remained depressed (PHQ-8 > 4 and no change in severity category) or worsened (change to a higher severity category) and 2) remained non-depressed (PHQ-8 ≤ 4 and no change in severity category) or improved (change to a lower severity category). The association between depression trajectories and VL non-suppression (HIV RNA > 200 copies/ml on the first measurement after a COVID-era survey) was assessed using logistic regression adjusting for age, gender, pre-pandemic VL, clinical diagnosis of mood and substance use disorders.

**Results:**

Among 793 PWH in this study, 60% were male, 88% were Black and the mean age was 56 years. Approximately 24% of PWH remained depressed (9%) or worsened (15%), while 76% remained non-depressed (60%) or improved (16%). PWH who remained depressed or worsened were more likely to be virally unsuppressed (adjusted odds ratio:2.45, 95% confidence interval: 1.19 – 5.06) compared to those who remained non-depressed or improved.

**Conclusion:**

In our cohort of PWH, a quarter either remained consistently depressed or experienced worsening depression in the COVID-era. Depression was significantly associated with VL non-suppression during the pandemic. Our findings suggest that strategies to monitor and address depression symptoms among PWH may contribute to reduced risk of VL non-suppression.

**Disclosures:**

**Oluwaseun Falade-Nwulia, MBBS ,MPH**, Abbvie Inc: Grant/Research Support|Gilead Sciences: Advisor/Consultant

